# Supplementary material for: Cause-specific student absenteeism monitoring in K-12 schools for detection of increased influenza activity in the surrounding community—Dane County, Wisconsin, 2014–2020
Source: PLoS One. 2022 Apr 19;17(4):e0267111. doi: 10.1371/journal.pone.0267111 (PMC9017898; doi:10.1371/journal.pone.0267111)
Supplement: Attachment — Submitted filename: Response to Reviewers letter.docx [file pone.0267111.s001.docx]

# **Editor’s Requests**

1. **The manuscript has been reformatted per the guidelines in the links provided.**

- <https://journals.plos.org/plosone/s/file?id=wjVg/PLOSOne_formatting_sample_main_body.pdf>
- <https://journals.plos.org/plosone/s/file?id=ba62/PLOSOne_formatting_sample_title_authors_affiliations.pdf>

2. **We have provided additional details regarding participant consent, as requested, pertaining to informed consent. We had added the following to the manuscript and submission form:**

“Written informed consent was obtained from parents/guardians of minor students and from students aged ≥18 years during home visits for collection or data and respiratory specimens as detailed in the ORCHARDS methodology.[15] All absenteeism data from the OSD’s information system was anonymous; no informed consent was needed per review by the Human Subjects Committees of the Education and Social/Behavioral Sciences IRB.”

3. **We have added additional information about the participant recruitment method, inclusion/exclusion criteria, and the demographic details (in table format) of participants as requested:**

“The full details pertaining to recruitment of students for data and specimen collection at home, including inclusion and exclusion criteria, are available in the ORCHARDS methods paper.[15] Inclusion criteria included: (1) student attends, or is eligible to attend (e.g., home schooled), a school within the OSD; (2) has an illness characterized by ≥2 of 6 acute respiratory infection/ILI symptoms (nasal discharge; nasal congestion; sneezing; sore throat; cough; fever); and (3) scores ≥2 points on the Jackson scale. Exclusion criteria included: (1) illness onset ≥7 days before anticipated time of specimen collection; (2) anatomical defect for which nasal specimen collection is contraindicated; and (3) student participated too recently (<7 days during peak influenza period and <30 days during other times, as determined by medically-attended surveillance program).

**Table 4: Characteristics of 2,378 ORCHARDS student participants in the home study.**

| Age, mean ± sd; [range] | 9.9 ± 3.5 years [4 – 18] |  |
| --- | --- | --- |
| Grades K-4 (4-9 years) | 1,202 (50.6%) |  |
| Grades 5-6 (10-11 years) | 418 (17.6%) |  |
| Grades 7-8 (12-13 years) | 352 (14.8%) |  |
| High School (14-18 years) | 406 (17.1%) |  |
| Sex |  |  |
| Female | 1,024 (43.2%) |  |
| Male | 1,349 (56.8%) |  |
| Time from symptom onset to home visit | 53.8 ± 32.3 hours |  |

4. **Revision of Figure 2:**

We modified Fig 2 to only include content that was either in the public domain or we received explicit permission to use. We received written permission to use the map of the Oregon School District and have included the requested form in our submission under “Other”. The map of Wisconsin is in the public domain and can be accessed here: <https://en.wikipedia.org/wiki/File:Map_of_Wisconsin_highlighting_Dane_County.svg>.

5. **Competing Interests section:**

We added a Competing Interest statement to the manuscript to explicitly state that Quidel Corporation provided in-kind materials. We also stated that this does not alter our ability to adhere to all PLOS ONE policies on sharing data and materials. The statement now reads as follows:

“JLT has received past research funding from Quidel Corporation. Quidel provided in-kind Sofia analyzers and Influenza A+B FIA tests to the Wisconsin research team. This does not alter our adherence to PLOS ONE policies on sharing data and materials. Quidel did not direct or exert any influence over study design, data collection and analysis, decision to publish, or preparation of the manuscript.”

6. **Dataset can be accessed via Harvard Dataverse**

<https://doi.org/10.7910/DVN/EGHXLS>

7. **We added a Supporting Information section in the manuscript. Captions are as follows:**
   - S1: First grant award
   - S2: Second grant award
   - S3: IRB exemption for absentee data collection
   - S4: IRB approval for first grant award
   - S5: IRB continuation for second grant award
   - S6: Content permission form for Fig 2

**Reviewer's Comments to the Author**

**Reviewer #1:**

*1. My main concern is regarding the methods of the paper. It says this is a prospective study. I am not sure how is it a prospective study. Would you please clarify it in the methods section?*

The reviewer appears to be envisioning a prospective cohort study, in which a single group of subjects is being followed over time, not the prospective monitoring of routinely collected school absence data. The full ORCHARDS methodology is long and complex and has been published as a stand-alone manuscript. We have emphasized the distinction between the data collection by the schools (the absenteeism data) and the home study data collection by research staff by adding the following at the beginning of the Materials and Methods section:

“ORCHARDS is a prospective, community-based study that captures school absenteeism data on a daily basis. In addition, school aged children with ARI are recruited to participant in a home study which allows evaluation of demographic, clinical, and virological correlates of respiratory infections.”

*2. Assuming it [ORCHARDS] is a prospective study, is the same individual were repeatedly participating in the survey?*

I am not sure what is meant here by the survey. We receive daily counts of absentee students in each of three categories of absenteeism. The data are anonymous. One would assume that some absent students are absent for more than one day, and, therefore, will have their absenteeism repeatedly recorded over more than one day. This constitutes the primary data set that is compared to daily counts of medically-attended influenza visits in surrounding communities using an independent surveillance system. There is no survey involved here.

Likewise, for our home study, we do allow for an individual to be visited more than once, but require this to be for a separate illness episode. Demographic, epidemiological, clinical and respiratory specimens are collected at the home visit. These data allow the validation of our definition of absenteeism due to influenza-like illness (a-ILI).

*3. Assuming it is a prospective study, please provide descriptive statistics of the baseline, final wave and pooled in all waves.*

The Wisconsin Department of Public Instruction determines each schools enrolled population once per year. As we point out in the text and Table 2, the student population in the Oregon School District grew marginally over the 6 years of the study (by about 1.3% per year). There are no missing data in the absenteeism data.

*4. Would you please report the regression results in tabular form?*

There are a large number of regression coefficients, none of which are directly interpretable. Unfortunately, given the brevity of the reviewer’s comment, it’s not really clear why they want a tabular presentation. Our statistician’s view was to possibly present the regression estimates at a regular series of timepoints, but that would likely be a very long table and would not be any more informative than the existing graphical representation of the model.

Accordingly, our statistician has generated a table presenting estimates from the GAM models (see following page for table; this could be included in the paper or provided as a supplement if so desired by the editor).  For the calendar day effect, he has presented the estimated mean/CI for the number of events on the first Monday of each month (no estimates are presented for absences when school is closed, mostly July and August, but occasionally January and April).

The MAI model/graphic actually nicely demonstrates how the model appropriately captures the very low rate of MAI outside of flu season, but the sporadic events outside flu season in most, if not all, years indicates that there’s never really a time when there’s zero risk of MAI.

*5. Would you please run Zero-inflated negative binomial regression and compare with the existing regression results?*

Our statistician has replied: “I strongly dislike zero-inflated models. They’re only appropriate in situations where it is impossible for the event of interest to occur on some days, but we don’t know exactly which days those are. The obvious application for our data would be if we didn’t know which days the schools were closed; there would be zero absences on those days for structural reasons, which could potentially produce a lot of excess zeros. Since we know when the schools were closed, we’ve already accounted for this. The other possibility would be that there are specific times when it is impossible, not just very unlikely, to have an event. I don’t think that applies to any of our outcomes. It’s possible to be absent, be absent due to illness, and be absent due to ILI at any time; you may be more likely to get ILI during flu season, but it’s not impossible to get an ILI (or even influenza) at any time.”

*6. Would you please provide a figure for the flow of the sample and missing data analyses?
I hope my comments will be helpful.*

We think this may reflect some confusion regarding the study design. There’s not a sample in the sense that the reviewer is talking about, and the only missing data issues would relate to participation in the home visits as there is no coupling between absenteeism and home visits. There are no missing data in the absenteeism data.

**Reviewer #2:**

*1. This work deals with the assessment of the relation between students' absenteeism and influenza activity (epidemics) in the surrounding communities. The data rely on a 6 -year observation of student attendance in the Oregon school district employing Infinite Campus system. The main conclusion is that there is a correlation between absenteeism due to illness (a-I) and particularly influenza-like illness (a-ILI) and influenza spikes. This observation can be used to mitigate influenza outbreaks. The work is sound and well written and potentially publishable. However, I find the a-ILI lead time rather short to be useful, while the a-I correlation seems relatively weak. Of course it is an improvement over a-TOT, but I somehow miss a strong practical message of immediate applicability, which makes me wonder if a more specialized epidemics journal would not be a better place for this work. Perhaps the authors can strengthen this point.*

The “lead times” refer to above are the points at which correlations achieve maximal levels. We do comment that significant correlations extend to a lead time of at least 14 days, which is of great significance when considering the serial interval of influenza (see lines 298—300 and 311—313):

(298-300) “For all six seasons, however, significant, positive cross-correlations were achieved for a-I and a-ILI at least 14 days in advance of MAI (Figure 5).”

(311-313) “Moreover, as time frames are shifted, significant correlations are demonstrated between cause-specific absenteeism (a-ILI and a-I) and MAI well in advance of MAI in all cases.”

We would also argue that the correlations are reasonably high, and in the case of significant influenza outbreaks, very high. This underscores the potential value for identification of significant influenza outbreaks, such as highly virulent and/or pandemic strains (see lines 332—336):

(332-336) “The strength of correlations for individual outbreaks of influenza appears to follow the intensity of the influenza season. The most significant influenza seasons experienced by the OSD and the surveillance clinics (2017-2018 and 2019-2020) resulted in very high maximal correlations (r=0.64 and 0.82, respectively) between a-ILI and MAI. This supports potential benefit of cause-based absenteeism in monitoring systems for pandemic influenza.”

*2. In the abstract the authors suggest that monitoring a-ILI and a-I can provide early warning, but isn't a 1 day lead time too short for early warning? I suggest making this point clear.*

A one-day lead time only refers to the maximal correlation achieved (see lines 298—300 and 311—313), as above. Again, there is an interplay between the strength of correlation and the lead time. This paper provides the empirical data that can be used to formulate appropriate thresholds for triggering alert systems.

(298-300) “For all six seasons, however, significant, positive cross-correlations were achieved for a-I and a-ILI at least 14 days in advance of MAI (Figure 5).”

(311-313) “Moreover, as time frames are shifted, significant correlations are demonstrated between cause-specific absenteeism (a-ILI and a-I) and MAI well in advance of MAI in all cases.”

*3. For the general audience of PLOS One, it will be useful if the authors briefly mention what kind of mitigation measures could be undertaken after a-I and a-ILI warning signals.*

We have added content to the last paragraph in the conclusions following “thus allowing for mitigation efforts” to now read: “thus allowing for mitigation efforts, such as use of face masks, physical distancing, testing, isolation, quarantining, and enhanced vaccination programs.”

*4. What is the reason for decreasing a-ILI and increasing a-TOT with grades? Are they related to medical/social issues?*

The most likely reason is that younger children are more prone to develop fevers with ARIs and that older children are more likely to be absent for a wide variety of reasons. We did not attempt to assess the causation of this pattern.

*5. Perhaps I missed it, but were (some) students also ARI patients?*

The absenteeism categories are for ILI (a subset of ARI), any illness, or any reason for absenteeism. ARI is requisite for a home visit, but ILI and absenteeism are not. The home visit component was used only to validate the definition of a-ILI and to assess the odds ratio of meeting a-ILI criteria if influenza is detected in a respiratory specimen.

*6. Figs 4 and 5 shows that the a-ILI and MAI spikes nearly coincide, which agrees with the 1-day lead time, questioning whether this can be used as an early warning signal to mitigate influenza. I suggest to discuss this issue when discussing these figures and also in the Conclusions.*

See previous comments pertaining to the lead times and correlations above.

*7. Why did the authors expect that dividing the data into K4 and K5-12 would have improved correlations?*

Younger children often have a more pronounced response to influenza and other respiratory viruses, than do older children, especially in regards to fever. This may be due to a lack of underlying, naturally acquired immunity. In addition, children from these five grades (out of 13) are represented in about 50% of our home visits. Finally, there is a natural cutoff in the Oregon School District as K-4 students are segregated into three schools, whereas the older age cohorts attend combined schools. We tested whether this age cutoff made any difference, so as to provide any additional guidance for districts that may implement this approach. It did not.
